# Heptapeptide Isolated from *Isochrysis zhanjiangensis* Exhibited Anti-Photoaging Potential via MAPK/AP-1/MMP Pathway and Anti-Apoptosis in UVB-Irradiated HaCaT Cells

**DOI:** 10.3390/md19110626

**Published:** 2021-11-09

**Authors:** Zhaowan Zheng, Zhenbang Xiao, Yuan-Lin He, Yanfei Tang, Lefan Li, Chunxia Zhou, Pengzhi Hong, Hui Luo, Zhong-Ji Qian

**Affiliations:** 1 School of Chemistry and Environment, Shenzhen Institute of Guangdong Ocean University, College of Food Science and Technology, Guangdong Ocean University, Zhanjiang 524088, China; zzwzhengzhaowan@163.com (Z.Z.); xzhenbang@163.com (Z.X.); heylzzz@163.com (Y.-L.H.); HYa0847@163.com (Y.T.); FLL@gdou.edu.cn (L.L.); chunxia.zhou@163.com (C.Z.); hongpengzhigdou@163.com (P.H.); 2 Southern Marine Science and Engineering Guangdong Laboratory, Zhanjiang 524025, China; luohui@gdmu.edu.cn; 3 The Marine Biomedical Research Institute, Guangdong Medical University, Zhanjiang 524023, China

**Keywords:** heptapeptide, photoaging, UVB, MMPs, apoptosis

## Abstract

Marine microalgae can be used as sustainable protein sources in many fields with positive effects on human and animal health. DAPTMGY is a heptapeptide isolated from *Isochrysis zhanjiangensis* which is a microalga. In this study, we evaluated its anti-photoaging properties and mechanism of action in human immortalized keratinocytes cells (HaCaT). The results showed that DAPTMGY scavenged reactive oxygen species (ROS) and increase the level of endogenous antioxidants. In addition, through the exploration of its mechanism, it was determined that DAPIMGY exerted anti-photoaging effects. Specifically, the heptapeptide inhibits UVB-induced apoptosis through down-regulation of p53, caspase-8, caspase-3 and Bax and up-regulation of Bcl-2. Thus, DAPTMGY, isolated from *I. zhanjiangensis*, exhibits protective effects against UVB-induced damage.

## 1. Introduction

Skin aging is an irreversible natural physiological process related to free-radical formation in the skin [1]. Under the current conditions of the Earth’s rotation, 280–3000 nm solar radiation reaches the Earth’s surface, among which the 280–1600 nm wavelengths can be divided into ultraviolet (UV), visible light (VIS), and near infrared (IR) [2]. However, UV radiation is the most concerning due to its much higher photon energy than VIS and IR [3]. Of the UV radiation that reaches the Earth’s surface, UVB (280–320 nm) is directly associated with photodamage and photoaging, as UVA (320–400 nm) confers approximately 1000-fold less photic damage than UVB [4]. Long-term exposure to UVB leads to skin wrinkling and collagen loss, which accelerates the premature aging of skin (so-called skin photoaging) [5,6]. The absorption of UVB can induce radical formation through photoionization. ROS are very reactive free-radical molecules involved in cellular damage and molecular signaling, and they play a crucial role in skin aging [4]. UVB-induced skin injury can lead to a large number of ROS-mediated signal transduction abnormalities, cell redox homeostasis imbalance, and excessive apoptosis. In addition, the UVB-mediated abnormal secretion of MMPs is also an important factor in the degradation of collagen. Whereas light damage can be self-repaired through epidermal thickening and the antioxidant system, this ability is limited in cases of severe damage [7,8]. Therefore, the use of various antioxidants to control UVB-induced damage is considered an important strategy.

Microalgae are autotrophic unicellular microorganisms present within both aquatic and terrestrial ecosystems [9]. They are widely applied as carbon sequestration agents [10], food supplements [11], feed [12], and biofuel [13], as well as in the wastewater treatment field [14]. Moreover, microalgae have been considered as specific sources of peptides which contain potentially untapped physiological functions and exert a positive impact by binding to specific receptors, interacting with target cells, or inhibiting enzymes [11]. It was reported that the skin gelatin hydrolysate of pacific cod showed inhibitory activity toward MMP-1 expression in mouse skin fibroblasts exposed to UV radiation [15]. Recent studies demonstrated that microalgae-derived biopeptides display antioxidant, antimicrobial, antitumor, and antihypertensive activities. For example, a hexapeptide from *Chlorella ellipsiodea* (Leu–Asn–Gly–Asp–Val–Trp) was shown to have antioxidant activities via scavenging peroxyl and DPPH radicals [16]. Another peptide isolated from *Tetraselmis suecica* exhibited bactericidal effects against both Gram-negative and Gram-positive human pathogenic bacteria [17]. In addition, a peptide byproduct from *Chlorella vulgaris* processing (Val–Glu–Cys–Tyr–Gly–Pro–Asn–Arg–Pro–Gln–Phe) was revealed to inhibit angiotensin I-converting enzyme (ACE) activity, thus exhibiting antihypertensive properties [18]. As presented above, the development and utilization of microalgae biopeptides is very promising.

There have been previous discoveries related to the activities of other peptides in *Isochrysis zhanjiangensis*. For example, peptides isolated from *I. zhanjiangensis* exhibited an antioxidant effect [19]. Chen et al. found that *I. zhanjiangensis* can inhibit angiotensin I-converting enzyme activity, thus exhibiting antihypertensive properties [20]. However, this research is neither extensive nor comprehensive; thus, there is a lack of accumulated results. In this study, we focused on whether a novel heptapeptide isolated from *Isochrysis zhanjiangensis* has anti-UVB-induced photoaging properties from the perspective of antioxidant and antiapoptotic activity and the inhibition of MMP secretion. As a result, the utilization value of *Isochrysis zhanjiangensis* can be improved, and the mechanistic activity of its isolated polypeptides can be supplemented through protein hydrolysis.

## 2. Results

### 2.1. UVB Stimulation and Effect of DAPTMGY on HaCaT Cell Viability

**Figure 1:**
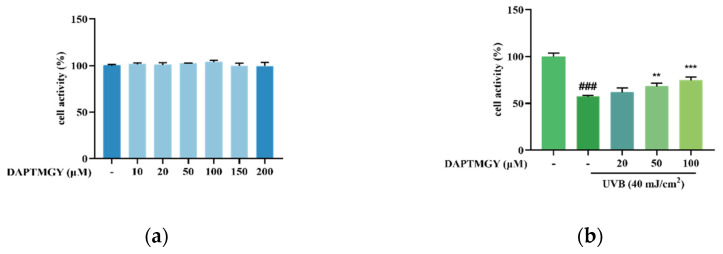
Effect of DAPTMGY on cell viability of HaCaT cells. (**a**) the viability of various doses of DAPTMGY (0, 10, 20, 50, 100, 150, and 200 µM) on HaCaT cells; (**b**) protective effects of DAPTMGY (0, 20, 50 and 100 µM) on UVB -induced (40 mJ/cm^2^) HaCaT cell injury. Data are shown as mean ± S.D (*n* = 3). (###) *p* < 0.001, as compared to the blank group (untreated cells). ** *p* < 0.01 and *** *p* < 0.001, respectively, as compared to the control group.

As shown in Figure 1a, after coculture with *DAPTMGY*, the viability of HaCaT cells revealed no significant change, demonstrating that the DAPTMGY concentration range of 1–200 µM is safe. Thus, 0, 20, 50, and 100 µM concentrations of DAPTMGY were used in the subsequent experiments. Cells were cultured with different DAPTMGY concentrations for 24 h (Figure 1b). The data indicated that DAPTMGY (50 and 100 μM) can effectively enhance cell viability in the presence of UVB.

### 2.2. Reducing Effect of DAPTMGY on Intracellularly Overexpressed ROS

**Figure 2:**
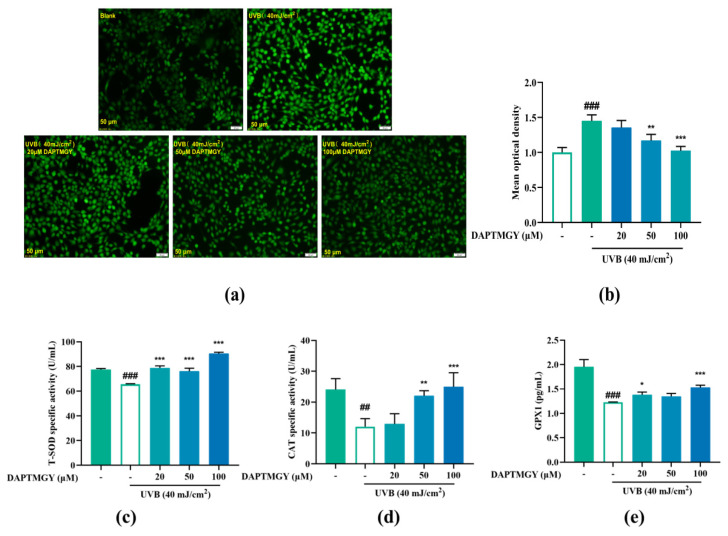
Effect of DAPTMGY on intercellular ROS generation and on the contents of Total superoxide dismutase (T-SOD), catalase (CAT) and glutathione peroxidase 1 (GPX1) in UVB-stimulated (40 mJ/cm^2^) HaCaT cells. Cells were cultured with DAPTMGY (20, 50, and 100 µM) for 2 h and then cultured for another 24 h after UVB irradiation. (**a**) Intercellular ROS production was measured in intact HaCaT cells using DCFH-DA, and photos were captured under fluorescence inverted microscopy. (**b**) The relative DCF fluorescence intensity analysis was conducted using Image J (fluorescence experiments were performed four times; *n* = 3 per group). (**c**–**e**) The levels of T-SOD, CAT, and GPX1 were measured using biochemical assay kits and ELISA kits. Results are shown as the mean ± SD (*n* = 3). ^##^ *p* < 0.01 and ^###^ *p* < 0.001 compared to the blank group (untreated cells); * *p* < 0.05, ** *p* < 0.01, and *** *p* < 0.001 compared to the control group.

Intracellular ROS changes were detected using the 2′,7′-dischlorodihydrofluorescein diacetate (DCFH-DA) fluorescent probe. Compared to the blank group, UVB irradiation led to significant fluorescence in HaCaT cells. However, as the concentration of DAPTMGY increased, the fluorescence signal gradually decreased (Figure 2a). Mean optical density analysis revealed that a relatively high concentration of heptapeptide could significantly reduce the ROS content (*p* < 0.01).

### 2.3. Regulatory Effect on Antioxidant Enzyme Activities

As shown in Figure 2c–e, compared to the UVB-stimulated group, the activities of T-SOD (*p* < 0.001), CAT (*p* < 0.01), and GPX1 (*p* < 0.05) were obviously enhanced after treatment with DAPTMGY. The results indicated that DAPTMGY can enhance the activities of endogenous antioxidant enzymes such as SOD, CAT, and GPX1.

### 2.4. Detection of Related Apoptosis Proteins

**Figure 3:**
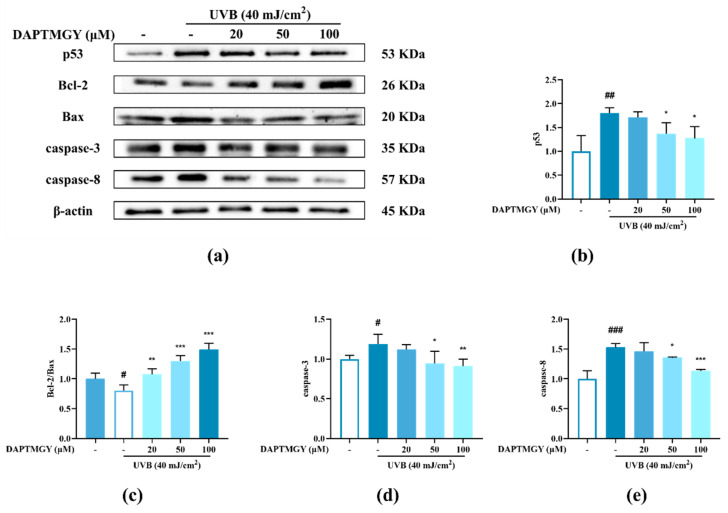

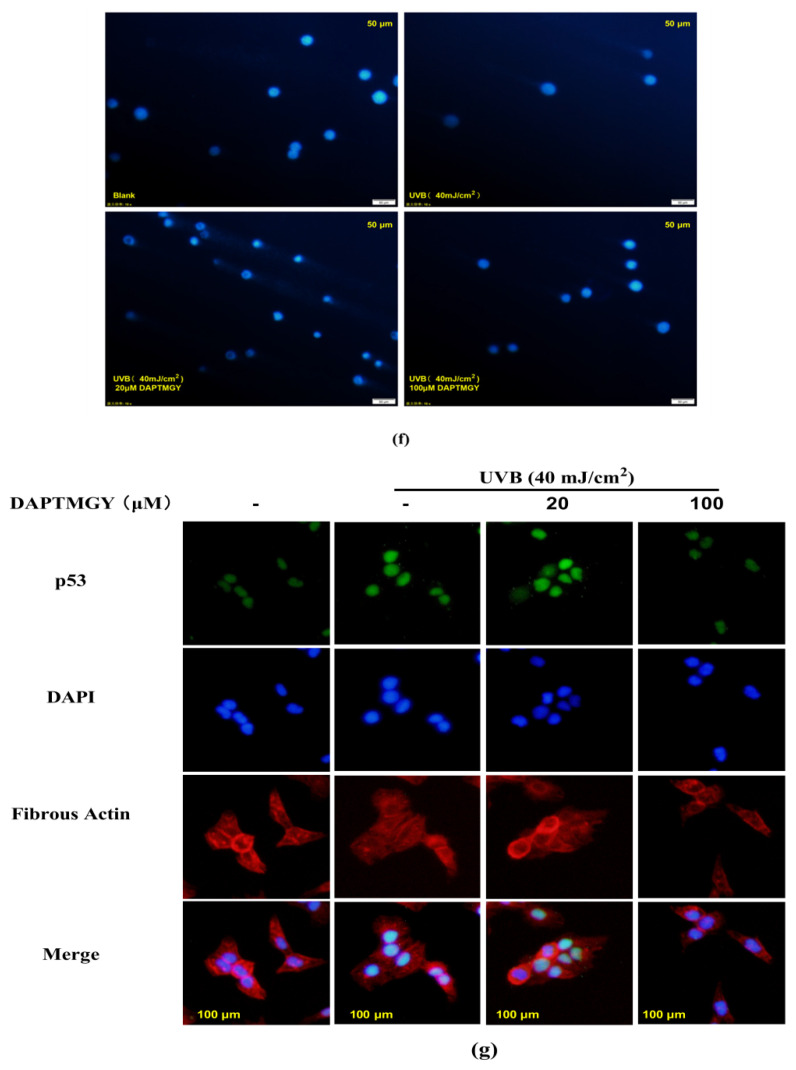
The influence of DAPTMGY on the levels of related apoptosis proteins in UVB-stimulated HaCaT cells. (**a**) Western blotting bands of p53, Bcl-2, Bax, caspase-3 and caspase-8. (**b**,**d**,**e**) The proteins expression of p53, caspase-3 and caspase-8, respectively. (**c**) The ratios of Bcl-2/Bax. (**f**) HaCaT cells stained with DAPI were observed using an inverted fluorescence microscope. (**g**) Nuclear translocation of p53 was observed by immunofluorescence through an overlay of green p53 staining, blue DAPI staining and Actin red. Data are shown as mean ± S.D (*n* = 3). (#, ## and ###) *p* < 0.05, *p* < 0.01 and *p* < 0.001, as compared to the blank group (untreated cells). * *p* < 0.05, ** *p* < 0.01 and *** *p* < 0.001, respectively, as compared to the control group. β-actin was used as an internal control.

**Figure 4:**
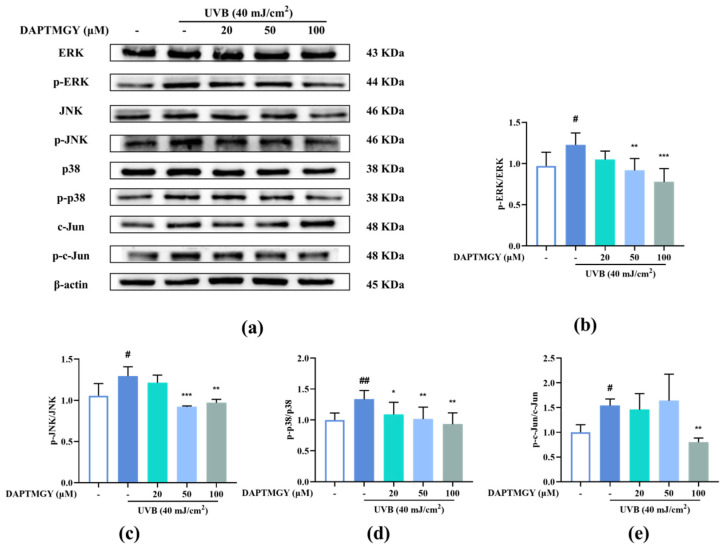
DAPTMGY blocked phosphorylation of ERK, p-38 and c-Jun in UVB-stimulated HaCaT cells. (**a**) Western blotting bands of MAPK pathway and AP-1. (**b**) The ratios of p-ERK/ERK, (**c**) The ratios of p-JNK/JNK, (**d**) The ratios of p-p38/p38 and (**e**) The ratios of p-c-Jun/c-Jun are calculated respectively. Data are shown as mean ± S.D (*n* = 3). (# and ##) *p* < 0.05 and *p* < 0.01, as compared to the blank group (untreated cells). * *p* < 0.05, ** *p* < 0.01 and *** *p* < 0.001, respectively, as compared to the control group. β-actin functioned as the internal control.

The expression of signaling pathways related to apoptosis, namely, Bax, Bcl-2, caspase-3, and caspase-8, was observed in UVB-exposed cells cultured with DAPTMGY (Figure 3). A significant upregulation of proapoptotic genes Bax, caspase-3, and caspase-8 was observed in the UVB + DAPTMGY group compared to the control group. Furthermore, an associated significant increase in antiapoptotic gene Bcl-2 was observed. Apoptosis depends on the activation of receptors in the mitochondrial-dependent death pathway, while the process is also affected by many other signaling pathways, such as p53 [21]. Therefore, the effect of DAPTMGY on p53 was evaluated (Figure 3). Western blot analysis showed that the level of p53 in the control group was significantly upregulated, whereas it was reduced after DAPTMGY treatment (Figure 3c). As shown in Figure 4a, after UVB exposure, p53 was significantly transferred into the nucleus. Subsequently, 100 µM DAPTMGY treatment effectively suppressed p53 nuclear translocation in HaCaT cells. As shown in Figure 3f, the comet tail of DAPTMGY at 100 µM concentration was shorter than that of the control group, indicating that a high concentration of DAPTMGY reduced the DNA damage induced by UVB.

### 2.5. DAPTMGY Regulates UVB-Induced MAPKs and AP-1 Activation

UVB irradiation upregulated the expression of p-c-Jun compared with the control group. However, treatment with DAPTMGY decreased the expression of p-c-Jun (Figure 4e). Compared with the blank group, the phosphorylation levels of ERK, JNK, and p38 were increased after UVB irradiation (Figure 4). Treatment with 20 µM DAPTMGY had no impact on the phosphorylated levels of ERK and JNK. However, the p-p38/p38 ratio was significantly decreased. At the concentration of 100 µM, it can be seen that the phosphorylation level of MAPK was significantly downregulated (Figure 4b–d), indicating that DAPTMGY can inhibit the signal pathways (MAPK and AP-1) related to photoaging.

### 2.6. Influence of DAPTMGY on NF-κB Signaling Pathway

**Figure 5:**
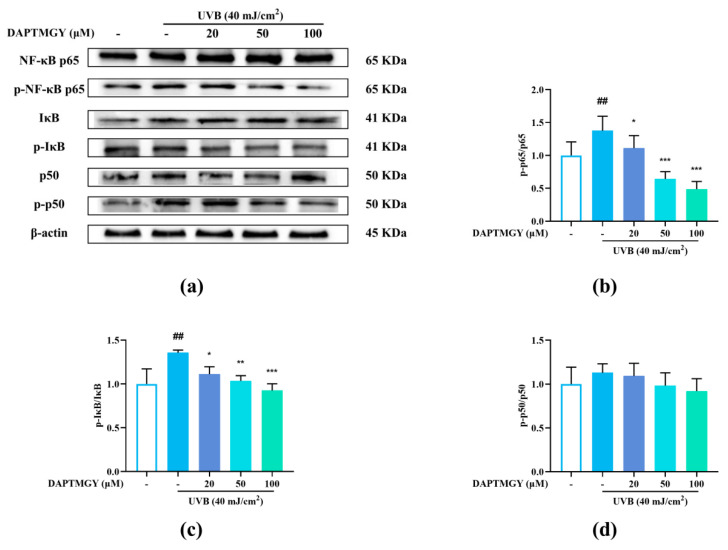
DAPTMGY suppressed the UVB-stimulated NF-κB signal pathway in HaCaT. (**a**) Western blotting bands of expression of NF-κB pathway. (**b**–**d**) The ratios of p-NF-κB p65/NF-κB p65, p-IκB/IκB and p-p50/p50 are calculated respectively. β-actin functioned as the control and all of the experiments were performed in at least triplicate. Data are shown as mean ± S.D (*n* = 3). (##) *p* < 0.01, as compared to the blank group (untreated cells). * *p* < 0.05, ** *p* < 0.01 and *** *p* < 0.001, respectively, as compared to the control group. β-actin functioned as the internal control.

Compared with the blank group, the phosphorylation levels of p65 and IκB were significantly increased after UVB stimulation. Contrarily, the p-p65/p65 and p-IκB/IκB ratios exhibited a dose-dependent decrease (Figure 5b,c), whereas the p-p50/p50 ratio showed no significant change (Figure 5d), indicating that DAPTMGY inhibited the NF-κB signaling pathway.

### 2.7. Expression Levels of MMP and Procollagen I Concentration

**Figure 6:**
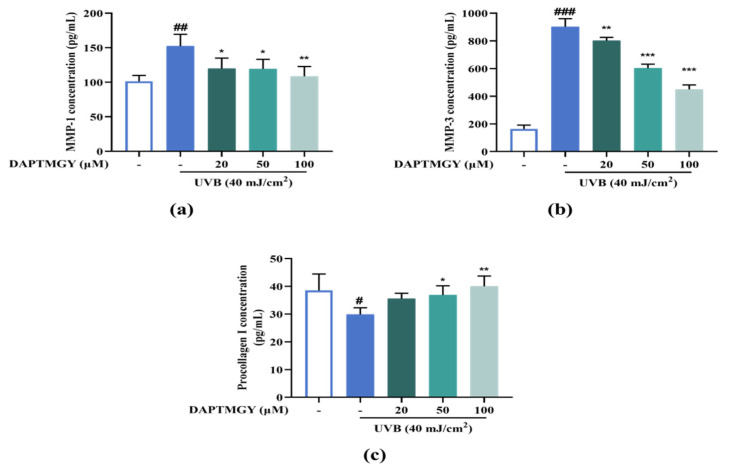
DAPTMGY suppresses MMP-1 and MMP-3 secretion and increase procollagen I. (**a**) The secretion in condition media of MMP-1, (**b**) The secretion in condition media of MMP-3, and (**c**) The secretion in condition media of procollagen I was assessed by ELISA kit. Data are shown as mean ± S.D (*n* = 3). (#, ## and ###) *p* < 0.005, *p* < 0.01 and *p* < 0.001, as compared to the blank group (untreated cells). * *p* < 0.005, ** *p* < 0.01 and *** *p* < 0.001, respectively, as compared to the control group.

UVB irradiation resulted in a significant increase in the secretions of MMP-1 and MMP-3 and a significant decrease in procollagen I compared with the control group. However, culture with 20–100 µM DAPTMGY decreased the MMP-1 and MMP-3 secretions and increased the content of procollagen I in HaCaT cells (Figure 6), evidencing the anti-photoaging activity of DAPTMGY.

### 2.8. Molecular Docking Analysis of MMP-1 and MMP-3 with DAPTMGY

**Figure 7:**
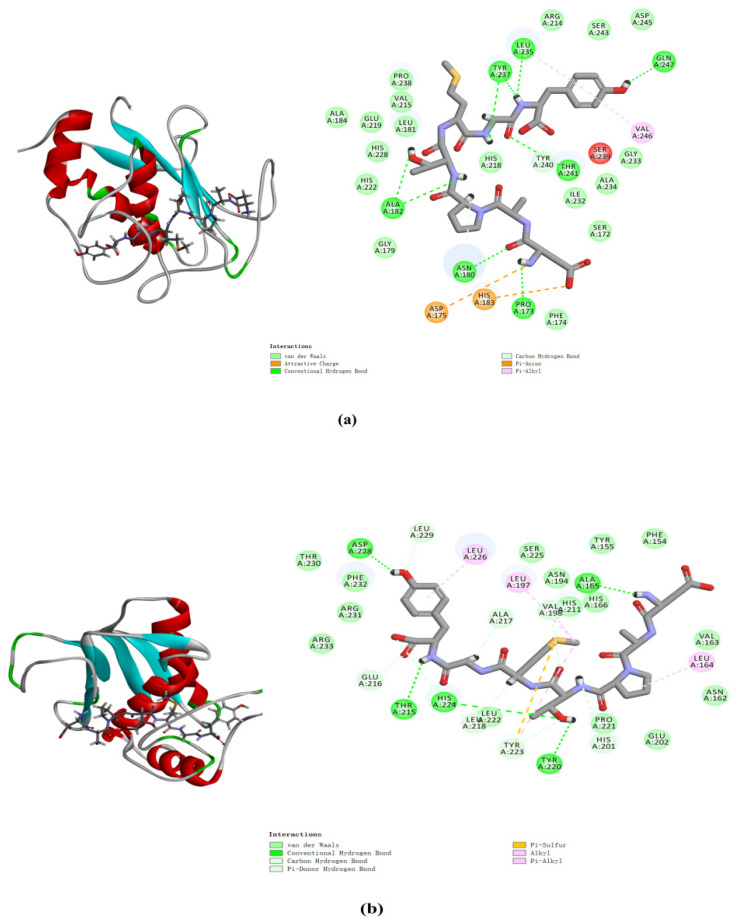
DAPTMGY interacted with MMP-1 (**a**) and MMP-3 (**b**). The optimal 3D poses structure and two-dimensional diagram of DAPTMGY and MMP-1 and MMP-3. Sticks stand for DAPTMGY and dotted lines is represent bond formation.

The docking simulation results revealed multiple interactions between DAPTMGY and MMP-1/MMP-3. These interactions were hydrophobic in nature, as clearly shown in Figure 7 and Table 1. The optimal CDOCKER interaction energy between DAPTMGY and MMP-1 and MMP-3 was −84.90 and −71.92 kcal/mol, respectively. DAPTMGY formed nine hydrogen bonds with MMP-1, including amino-acid residues Pro173, Asn180, Ala182 (two bonds), Leu235, Tyr237 (two bonds), Thr241, and Gln247. On the other hand, it formed five hydrogen bonds with MMP-3, including amino-acid residues Ala165, Thr215, Tyr220, His224, and Asp228. In addition, two pi–anion bonds and four pi–alkyl bonds were formed with MMP-1, while one pi–sulfur bond and six pi–alkyl bonds were formed with MMP-3.

## 3. Discussion

Active peptides often show good biological activity at low doses, which is an important feature of bioactive peptides [22]. The results of this study showed that DAPTMGY exhibited better protection against phototoxicity, as well as biocompatibility, in the concentration range of 20–100 µM. It can be seen that heptapeptide DAPTMGY isolated from *Isochrysis zhanjiangensis* has good potential as an anti-photoaging agent. The anti-photoaging protective mechanism was further explored with respect to oxidative stress and cell apoptosis.

Skin exposure to UVB activates oxidative stress of the epidermis [23]. On the one hand, under oxidative stress, excess ROS could overwhelm endogenous antioxidants (T-SOD, CAT, GPX1, etc.), leading to irreversible accumulation in cells [24,25], thus damaging the mitochondrial structure, collapsing the mitochondrial membrane potential, and initiating key apoptotic events. In this research, the generation of ROS was enhanced by UVB stimulation. Interestingly, DAPTMGY treatment could reduce ROS production, as well as increase the intracellular activities of T-SOD, CAT, and GPX1, thereby contributing to improved redox balance in UVB-damaged cells. To deduce its source of antioxidant activity, the composition of DAPTMGY was analyzed. Asp at the N-terminus of the heptapeptide can prevent the oxidation of unsaturated fatty acids [26] and is often used as a stabilizer of vitamin E in cosmetics and medicines. In addition, Asp–Ala may be a key antioxidant amino-acid fragment. The peptide Ala–Asp–Ala–Phe with the highest antioxidant activity was identified for the first time from walnut protein hydrolysate [27]. Byproducts from the hydrolysis of tuna (Trp–Gly–Asp–Ala–Gly–Gly–Try–Try) exhibited good antioxidant activity [28], with the phenolic hydroxyl group of Tyr at the C-terminus being an excellent hydrogen donor [29]. Moreover, the presence of the sulfur-containing amino acid Met strengthens the antioxidant properties of peptides [30]. It has been reported that methionine is widely applied in the medical care, animal breeding, and food additive industries [31]. The existence of these amino acids likely contributed to the good antioxidant capacity of DAPTMGY.

On the other hand, as a critical signal intermediate, ROS activates the expression of apoptotic genes and leads to apoptosis [32]. Controlled by Bcl-2 family proteins, death receptors, caspases, etc., cell apoptosis is primarily divided into exogenous and endogenous pathways [33]. In the exogenous pathway, death receptors are clustered and internalized in keratinocytes after UVB irradiation, resulting in caspase-8 initiation. Subsequently, the apoptosis signal is amplified by executioner caspases-3 in the cells. The results of this study are consistent with this mechanism. The intrinsic pathway involved in UVB-induced apoptosis results from DNA damage and further generates intracellular ROS [34]. This process is mainly orchestrated by Bcl-2 family members, which comprise antiapoptotic and proapoptotic proteins. It is worth noting that the ratio of anti- and proapoptotic Bcl-2 family proteins (Bcl-2/Bax), which modulate the mitochondrial pathway, appears to determine the sensitivity to apoptotic stimuli, as opposed to the expression of a single protein [35]. ROS lead to DNA damage in cells [36], resulting in p53 accumulation in the nucleus, which directly regulates the expression of proapoptotic gene Bax. p53 also accumulates in the cytoplasm, where it directly binds to Bcl-2 and interacts with the mitochondrial-mediated pathway to regulate apoptosis [37]. Furthermore, Bcl-2 can also activate Bax under signals inducing apoptosis, whereby Bax is oligomerized and inserted into the outer mitochondrial membrane [38]. Subsequently, mitochondria leakage of cytochrome C activates caspase-3 and induces apoptosis [39]. In our study, DAPTMGY was demonstrated to decrease the expression of p53 in the nucleus and concomitantly enhance the expression of Bcl-2 proteins, along with a decrease in Bax protein expression in HaCaT cells. Therefore, a shift in the Bax/Bcl-2 ratios prevented mitochondrial-mediated apoptosis. The results show that DAPTMGY significantly inhibited expression of the caspase cascade (caspases-3 and -8), resulting in the inhibition of apoptosis. p53 is not only a transcriptional factor, but also a major sensor of DNA damage, thus acting as an important regulator of DNA repair [40]. Normal cells irreversibly exit the cell cycle (senescence) upon telomere attrition and DNA damage [41]. In UVB-irradiated cells, DNA damage activates p53 and leads to cell-cycle arrest, leading to rapid aging and excessive apoptosis [42]. Moreover, UVB exposure leads to tissue damage through apoptosis [43]. Therefore, the upregulation of p53 as a cell-cycle inhibitor can induce apoptosis in response to DNA damage [44]. Moreover, DAPTMGY downregulated the expression level of p53 in the nucleus, ultimately inhibiting cell apoptosis and preventing photodamage. Therefore, our research proves that DAPTMGY can inhibit apoptosis through the intrinsic and extrinsic pathways. In addition, the overexpression of MMPs is the key to destroying the extracellular matrix (ECM) and leading to a loss of skin elasticity and a reduction in collagen. Oxidative stress can disrupt the balance between the accumulation and degradation of ECM components that support skin tissue structure and function; hence, MAPKs (ERK, JNK, and P38) and NF-κB (p50 and p65) that regulate oxidative stress [45] can regulate the gene expression of MMP-1 and MMP-3 in photoaging [46]. The UV-induced excess of intracellular ROS activates MAPKs and NF-κB, culminating in the transcriptional regulation of MMPs, especially MMP-1 and MMP-3. Additionally, the transcription factor AP-1 controls the expression of MMPs through the MAPK signaling pathway and inhibits transforming growth factor β signaling, caused a decrease in procollagen synthesis [47,48]. This leads to the degradation of collagen and elastin, which is an important sign of photoaging. According to the Western blot results, the levels of phosphorylated ERK, JNK, and p38 decreased after DAPTMGY treatment. MAPKs were activated, thus triggering NF-κB activation. In this study, the ratios of p-NF-κB, p65/NF-κB, p65, and p-IκB/IκB increased, whereas the ratio of p-p50/p50 did not change significantly. ELISA revealed that UVB irradiation leads to the overexpression of MMPs; however, DAPTMGY can protect against collagen degradation to some extent (Figure 5a,b).

Molecular docking studies have been helpful in earlier research to explore protein–ligand interactions [49,50]. In order to explore the interaction between DAPTMGY and MMPs, the active-site residues of MMP-1 and MMP-3 models were kept flexible and then docked with the DAPTMGY ligand to explore possible pathways for the inactivation of these proteins. The structures of MMPs are similar, with the main differences occurring in their hydrophobic domains (S1’ pocket), whereby many inhibitors typically occupy the S1’ pocket [51]. MMPs and DAPTMGY formed many interactions (pi–anion, pi–alkyl, pi–sulfur, etc.), with hydrogen bonding being the predominant contributor [52,53]. Our results show that DAPTMGY formed nine hydrogen bonds with MMP-1 and five hydrogen bonds with MMP-3 residues (Table 1), including with the polar atoms of amino-acid residue Thr241 in the S1′ pocket and Asn180 in the S3′ pocket. Moreover, the hydroxyl groups of DAPTMGY formed shorter hydrogen bonds with Gln247 and ionic interactions with the polar nitrogen atom of Ala182. Therefore, this study suggests that the inhibition of MMP-3 by DAPTMGY may be mediated by hydrogen bonding with Ala165 in the S1 pocket. In addition, DAPTMGY could also interact with the MMP-1 active site. The inactivation of these MMPs would lead to a reduction in their expression.

DAPTMGY holds promising potential as an anti-photoaging drug substitute for UVB-induced damage with antiapoptotic effects; however, subsequent in vivo animal experiments, evaluations in other skin cells, and pharmacokinetic analyses are required to further confirm its effects.

## 4. Materials and Methods

### 4.1. Chemicals and Materials

Dulbecco’s modified eagle’s medium (DMEM), fetal bovine serum (FBS), trypsin-EDTA (0.25%) and penicillin/streptomycin were purchased from Gibco (New York, NY, USA). Cell Counting Kit was produced by ZETA LIFE Inc. (Menlo Park, CA, USA). 4′,6-diamidino-2-phenylindole (DAPI), 2,7-dichlorodihydroflfluorescein diacetate (DCFH-DA) was obtained from Sigma-Aldrich (St. Louis, MO, USA). ELISA kits of MMP-1 (E-EL-H6073), MMP-3 (E-EL-H1446c), procollagen I (E-EL-H0181c) and Glutathione peroxidase 1 (GPX1, E-EL-H5410c) were purchased from Elabscience Biotechnology Co., Ltd. (Wuhan, China). Total superoxide dismutase (T-SOD, E-BC-K019-M) and Catalase (CAT, E-BC-K031-M) were purchased from Elabscience Biotechnology Co., Ltd. (Wuhan, China). P-c-Jun (9261), c-Jun (9165), Bcl-2 (3498), Bax (2772) and c-3 (14,220) were provided by Cell Signaling Technology (CST, MA, USA). p65 (sc8008), p-p65 (sc-136548), p50 (sc-8414), p-p50 (sc-271908), IκB-α (sc-1643), p-IκB-α (sc-8404), ERK (sc-94), p-ERK (sc-81492), JNK (sc-7345), p-JNK (sc-6254), p38 (sc-535), p-p38 (sc-166182), p53 (sc-126), c-8 (sc-5263), β-actin (47,778), antibodies (goat anti-rabbit IgG-HRP, sc-2004; goat anti-mouse IgG-HRP, sc-2005) were provided by Santa Cruz Biotechnology Inc. (Santa Cruz, CA, USA). All other drugs and solvents used are analytical grade.

### 4.2. Amino Acid Sequence Identification of DAPTMGY

**Figure 8:**
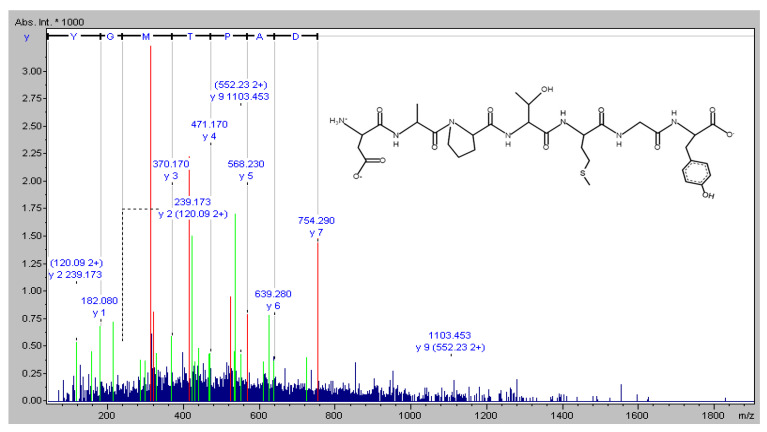
The amino acid sequence of DAPTMGY.

The novel peptide was isolated from *Isochrysis zhanjiangensis* hydrolysate obtained in our previous study [19]. The molecular mass of the peptide was analyzed, and the amino-acid sequence was determined using a Synchropak RPP-100 analytical column (4.6 × 250 mm) and a Q-TOF mass spectrometer coupled with an electrospray ionization (ESI) source (Micromass, Altrincham, UK). Its molecular mass was evaluated by mass spectroscopy in the double-charged state [M + 2H]^2+^ (data not shown). The amino-acid sequence was plotted using pepdraw (http://www.tulane.edu/~biochem/WW/PepDraw/, accessed on 5 October 2021) (Figure 8).

### 4.3. Cell Culture

Human immortalized keratinocytes (HaCaT) cells were bought from Shanghai IBS cell resource center. HaCaT cells were incubated (37 °C, 5% CO_2_) in DMEM (12% FBS and 1% penicillin/streptomycin).

### 4.4. UVB Irradiation and DAPTMGY Treatment

Cells grown to a suitable density were treated with different concentrations of DAPTMGY (20, 50, and 100 µM) for 2 h, washed with phosphate-buffered saline (PBS), and exposed to UVB light using a Philips PL-S 9W/01 source (peak emission at 311 nm; Royal Dutch Philips Electronics Ltd., Amsterdam, Holland). In this study, 40 mJ/cm^2^ was selected as the radiation dose. The blank group was treated with serum-free medium and no UVB irradiation, whereas the control group was treated with UVB irradiation without DAPTMGY; the remaining groups were treated with different concentrations of DAPTMGY. The cells and supernatants were used for subsequent experiments after 24 h.

### 4.5. Determination of Cytotoxicity

Cytotoxicity was assessed using the CCK-8 assay. Cells were seeded at 1 × 10^4^ cells/well in 96-well plates, exposed to UVB irradiation, and treated with different concentrations of DAPTMGY. Following 24 h incubation, 10 µL of CCK was added, and the reaction occurred at 37 °C. Next, the absorbance of each well was detected after 1 h at 450 nm using a microplate reader (BioTek, Winooski, VT, USA).

### 4.6. Content of Intracellular ROS

After treatment with different concentrations of DAPTMGY and irradiation with 40 mJ/cm^2^ UVB, HaCaT Cells were processed using a DCFH-DA fluorescent probe, kept away from light for 30 min at 37 °C, and then washed with PBS three times. Treated cells were observed using an inverted fluorescence microscope (Olympus, Tokyo, Japan).

### 4.7. Western Blotting

An equal amount of protein was subjected to SDS-PAGE before transferring to PVDF membranes. Subsequently, the membranes were blocked by 7% defatted milk for 2 h before adding primary antibodies overnight at 4 °C. Then, they were washed three times with TBST before binding to secondary antibodies for 2 h. Finally, the results were evaluated by enhanced chemiluminescence.

### 4.8. Analysis of Intracellular T-SOD, CAT and GPX1

Samples of different concentrations and cells irradiated by UV were scraped and placed in a 1.5 mL centrifuge tube. Then, the supernatant was extracted after centrifugation at 13,000 rpm for 10 min at 4 °C. The supernatant underwent three freeze–thaw cycles. The activities of T-SOD, CAT, and GPX1 were determined using biochemical assay kits according to the manufacturer’s recommended protocol.

### 4.9. Enzyme-Linked Immunosorbent Assay of MMP-1, MMP-3 and Type I Procollagen

The secretions of MMP-1, MMP-3, and type I procollagen in cellular supernatants were detected by commercially available ELISA kits (Elabscience, Wuhan, China). Cells irradiated by UVB were treated with different concentrations of DAPTMGY (0, 20, 50, and 100 µM) for 24 h. Conditional medium or a solution containing cell lysates was harvested in tubes and centrifuged (12,000 rpm, 4 °C, 10 min) to obtain the supernatant. The contents of MMP-1, MMP-3, and procollagen I were determined according to the manufacturer’s recommended protocol. The absorbance of the supernatant was measured under the condition of 450 nm wavelength of the microplate reader, and the standard curve was taken to calculate the level of MMP-1, MMP-3, and type I procollagen.

### 4.10. Comet Assay

Cells grown to a suitable density were treated with DAPTMGY at various concentrations for 2 h. After 24 h, the cells digested by trypsin were centrifuged before discarding the supernatant and resuspending the pellet in PBS. Subsequently, a comet assay was conducted according to the method of Lin et al. [54]. The stained nucleus was observed using an inverted fluorescence microscope (Olympus, Tokyo, Japan).

### 4.11. Immunocytochemistry

Treated cells were washed three times with PBS and fixed in 4% paraformaldehyde phosphate-buffered solution (4 °C, 30 min). The cells were permeabilized with 0.2% Triton X-100 (20 min) on ice and blocked with 5% bovine serum albumin (BSA, 1 h) at room temperature, followed by incubation with p53 antibody (1:500, 4 °C) overnight. After washing by PBS, the cells were bathed in goat anti-mouse IgG Dylight 488 (1:500; 2 h) and Actin red (1:100, 1 h) at room temperature. The nuclei were stained using 4′,6-diamidino-2-phenylindole (DAPI) and then viewed using an inverted fluorescence microscope (Olympus Opticals, Tokyo, Japan).

### 4.12. Molecular Docking

The structures of MMP-1 (966c.pdb) and MMP-3 (2JT6.pdb) were obtained from the Protein Data Bank. The docking method and steps were consistent with a previous study [55], and the best 3D docking pose was visualized.

### 4.13. Statistical Analysis

GraphPad Prism 8 (GraphPad Software, San Diego, CA, USA), CAPS (Version 1.2.3 beta1, Krzysztof Konca, url: CaspLab.com, accessed 1 February 2021), and Image J (Version 1.46r, NIH) were used for data analyses. One-way analysis of variance (ANOV A) was applied, and the differences between two groups were analyzed using Dunnett’s multiple comparison test. All experiment data were recorded in triplicate and expressed as the mean ± standard deviation; significance was assessed as * *p* < 0.05, ** *p* < 0.01, and *** *p* < 0.001.

## 5. Conclusions

**Figure 9:**
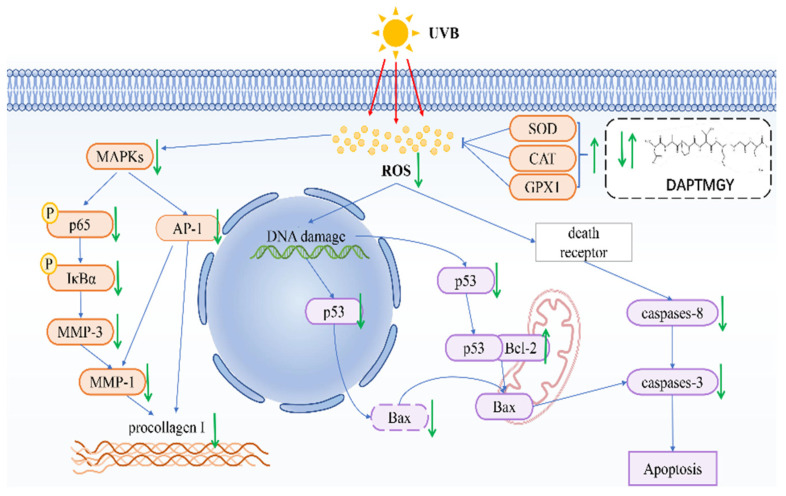
Mechanism summary of DAPTMGY against UVB-irradiated HaCaT cells oxidative and apoptosis.

In conclusion, we identified the amino-acid sequence of DAPTMGY and examined its anti-photoaging properties, as well as its underlying mechanism of inhibitory action, through in vitro assays and molecular docking analysis. Treatment with DAPTMGY attenuated UV-induced photoaging in HaCaT cells through suppression of ROS generation, apoptosis signaling transduction, and MMP expression (Figure 9). Thus, this study identified DAPTMGY as a novel peptide from marine microalgae, *I. zhanjiangensis* with potential to inhibit photoaging.

## Figures and Tables

**Table 1:** Results of DAPTMGY docking with MMP-1 and MMP-3, respectively.

DAPTMGY	Interaction Energy (-kcal/mol)	Hydrogen Bond Number	Interaction Atoms	Distance (Å)
MMP-1(966C)	84.90	9	Pro173	1.74
			Asn180	2.09
			Ala182	2.05
			Leu235	3.09
			Tyr237	2.45
			Thr241	2.31
			Gln247	1.96
MMP-3(2JT6)	71.92	5	Ala165	1.89
			Thr215	2.28
			Tyr220 His224 Asp228	2.86 3.08 1.85

## Data Availability

All data is contained within this article.
